# Cardiac and Peripheral Autonomic Responses to Orthostatic Stress During Transcutaneous Vagus Nerve Stimulation in Healthy Subjects

**DOI:** 10.3390/jcm8040496

**Published:** 2019-04-11

**Authors:** Eleonora Tobaldini, Edgar Toschi-Dias, Liliane Appratto de Souza, Karina Rabello Casali, Marco Vicenzi, Giulia Sandrone, Chiara Cogliati, Maria Teresa La Rovere, Gian Domenico Pinna, Nicola Montano

**Affiliations:** 1 Department of Internal Medicine, Fondazione IRCSS Ca’ Granda, Ospedale Maggiore Policlinico, 20122 Milan, Italy; eleonora.tobaldini@unimi.it (E.T.); edgardias@usp.br (E.T.-D.); 2 Department of Clinical Sciences and Community Health, University of Milan, 20122 Milan, Italy; marco.vicenzi@unimi.it; 3 Heart Institute (InCor), University of Sao Paulo Medical School, Sao Paulo 01246-903, Brazil; 4 Institute of Cardiology of Rio Grande do Sul, Porto Alegre 90040-060, Brazil; li.appratto@gmail.com; 5 Institute of Science and Technology, Federal University of Sao Paulo, Sao Paulo 04021-001, Brazil; krabello@terra.com.br; 6 Department of Cardiology, Fondazione IRCSS Ca’ Granda, Ospedale Maggiore Policlínico, 20122 Milan, Italy; 7 Department of Internal Medicine, ASST Fatebenefratelli-Sacco, 20131 Milan, Italy; giulia.sandrone@asst-fbf-sacco.it (G.S.); chiara.cogliati@asst-fbf-sacco.it (C.C.); 8 Department of Cardiology and Biomedical Engineering, Istituti Clinici Scientifici Maugeri, Istituto di Ricovero e Cura a Carattere Scientifico (IRCCS), Istituto di Montescano, 27040 Pavia, Italy; mariateresa.larovere@icsmaugeri.it (M.T.L.R.); giandomenico.pinna@fsm.it (G.D.P.)

**Keywords:** transcutaneous vagus nerve stimulation (tVNS), neuromodulation, cardiac autonomic control, orthostatic stress, symbolic analysis

## Abstract

Previous studies showed that transcutaneous vagus nerve stimulation (tVNS) modulates the autonomic nervous system (ANS) in resting condition. However, the autonomic regulation in response to an orthostatic challenge during tVNS in healthy subjects remains unknown. We tested the hypothesis that tVNS reduces heart rate (HR) and alters the responsivity of ANS to orthostatic stress in healthy subjects. In a randomized and cross-over trial, thirteen healthy subjects underwent two experimental sessions on different days: (1) tVNS and (2) control. Using a tVNS device, an auricular electrode was placed on the left *cymba conchae* of the external ear; an electric current with a pulse frequency of 25 Hz and amplitude between 1 and 6 mA was applied. For the assessment of ANS, the beat-to-beat HR and systolic arterial pressure (SAP) were analyzed using linear and nonlinear approaches during clinostatic and orthostatic conditions. In clinostatic conditions, tVNS reduced HR (*p* < 0.01), SAP variability (*p* < 0.01), and cardiac and peripheral sympathetic modulation (*p* < 0.01). The responsivity of the peripheral sympathetic modulation to orthostatic stress during tVNS was significantly higher when compared to the control session (*p* = 0.03). In conclusion, tVNS reduces the HR and affects cardiac and peripheral autonomic control and increases the responses of peripheral autonomic control to orthostatic stress in healthy subjects.

## 1. Introduction

The sympathetic and parasympathetic nervous systems are the main branches of the autonomic nervous system (ANS), and they dynamically control the visceral functions to maintain body homeostasis [1]. A hallmark of ANS is its great ability to react to environmental challenges/stressors (i.e., postural change) in order to properly respond to the metabolic demands of the organism [1,2].

Due to gravitational effects on body fluid distribution, several mechanisms are involved in maintaining cardiac output during orthostatic stress [3,4]. Peripheral vasoconstriction and increased heart rate (HR) are the major cardiovascular responses to postural change. These modifications are part of the reflex response elicited by negative feedback mechanisms (e.g., arterial baroreflex and cardiopulmonary reflex) [3,4]. Thus, with the fall of venous return, parasympathetic modulation decreases while sympathetic vasomotor activity increases progressively with the angle of body inclination [3,4].

Autonomic dysfunction plays a key role in the onset and progression of many diseases, such as hypertension and heart failure [5,6] and it has been shown that it is an independent prognostic factor for adverse cardiovascular outcome [7,8]. In addition, autonomic responses to orthostatic stress have clinical implications in cardiovascular disease [9,10]. In the study of Folino et al. [10], heart failure patients with blunted autonomic responses to orthostatic stress showed poor prognosis [10]. Thus, the ability of the ANS to react to evocative stimuli (e.g., orthostatic maneuver) can indicate a better prognosis in patients with heart failure.

A huge amount of literature has documented the beneficial effects of pharmacological and non-pharmacological treatments aimed at reducing the hyperadrenergic state in cardiovascular diseases, such as beta-blocker therapy and exercise training, respectively [11,12,13,14]. However, very few options are available to increase vagal modulation, mostly non-pharmacological treatments (such as yoga and respiratory-based techniques) [15,16]. In this sense, the possibility to directly and non-invasively modulate the vagus nerve using transcutaneous vagus nerve stimulation (tVNS) seems to be a promising therapy for cardiovascular and non-cardiovascular disorders [17,18].

It has been documented that the auricular branch of the vagus nerve of humans projects to the nucleus tract solitarius (NTS), which is the first central relay of vagal afferents, and to other vagal projections in the brainstem and forebrain [19]. By means of neuroimaging studies using functional magnetic resonance, Kraus et al. observed that BOLD signal in autonomic neuroregulatory pathways, as limbic structures and the brainstem, decreases during electrical stimulation of the left anterior auditory canal [20].

To our knowledge, few studies have assessed the effects of tVNS on the autonomic nervous system in healthy individuals [21,22,23]. In the study by Clancy et al., tVNS acutely reduced efferent sympathetic nerve traffic and sympathovagal balance [22]. In addition, Antonino et al. also observed a reduction of cardiac sympathovagal balance, likely due to improved arterial baroreflex control of HR [21]. However, the response of autonomic branches to an orthostatic challenge during tVNS in healthy subjects remains unknown. Thus, we tested the hypothesis that tVNS reduces HR and alters the responsivity of the autonomic nervous system to orthostatic stress in healthy subjects.

## 2. Materials and Methods

### 2.1. Subjects

Thirteen young healthy subjects (5 males, 8 females) with a mean age of 27 ± 4 years were recruited. Inclusion criteria were an age greater than 18 years and a stable sinus rhythm on the electrocardiogram (ECG). Exclusion criteria were a history of any known disease, any ongoing pharmacological treatment, and active smoking. Subjects were asked not to drink caffeine or alcohol within the 12 h before the experimental sessions.

In this randomized and cross-over study design, all subjects underwent a two-day protocol, one day with tVNS and a control day, at least 24 h after the first day. To evaluate cardiac and peripheral autonomic control, we continuously recorded the following cardiovascular signals: ECG (derivation II), respiratory signal through a thoracic belt (BT 16 Plus, FM Elettronica, Monza, Italy), and non-invasive beat-to-beat arterial blood pressure (Finometer MIDI®, Finapres Medical System^®^, Amsterdam, The Netherlands) at rest and during a passive orthostatic maneuver (tilt test 75°). During the experiments, subjects were in spontaneous breathing, but they were not allowed to talk. The signals were recorded with a sampling frequency of 1000 Hz for ECG and respiratory signal, while beat-to-beat arterial blood pressure was sampled at 250 Hz [24].

Therefore, on the tVNS day, the signals were recorded for 10 min in the supine position with the stimulator switched off (rest_tVNS off), 10 min in the supine position with the stimulator switched on (rest_tVNS on), and for 15 min in the orthostatic position with tVNS on (tilt_tVNS on). On the control day, signals were recorded for 10 min in supine position (rest_control) and 15 min in orthostatism (tilt_control). The percentage of changes to orthostatic stress (Δ%, tilt-test vs. resting condition) during tVNS or control days were used to evaluate the response of cardiac and peripheral autonomic control. The protocol was approved by the ethics committee of L. Sacco Hospital (Milan, Italy) and it was developed in accordance with the Declaration of Helsinki. All subjects signed informed written consent to the study.

### 2.2. Stimulation Procedure

As previously described [25,26], the transcutaneous electrical nerve stimulation was performed using a noninvasive TENS device (NEMOS®; Cerbomed, Erlangen, Germany) in the left *cymba conchae* of the external ear. Throughout the surface electrodes, an electrical current was applied continuously with a pulse width of 200 ms and pulse frequency of 25 Hz. The stimulation amplitude was adjusted to between 1 and 6 mA, at a level of each participant’s sensory threshold, until a comfortable sensation without pain was reported during stimulation [25,26].

### 2.3. Cardiac and Peripheral Autonomic Control

*Spectral analysis.* A linear method to assess the cardiovascular variabilities was used by means of specific software (HeartScope II; AMPS-LLC, New York, USA) on a personal computer by a trained investigator (ETD). Time series of the heart rate (tachogram) were obtained from the interval between two consecutive peaks of the R–R interval. Time series of systolic blood pressure (systogram) were generated by detection of the systolic peaks of blood pressure. In stationary sequences, segments of 200–300 beats were selected and analyzed by an autoregressive frequency domain approach [27,28,29]. Frequency domain analysis of heart rate variability (HRV) and systolic arterial pressure variability (SAPV) was performed with emphasis on low (LF: 0.04–0.15 Hz) and high frequency (HF: 0.15–0.40 Hz). The spectral components were expressed in absolute (abs.) and normalized units (n.u.). It is important to point out that LF is not an index of sympathetic activity. On the contrary, it is an index of sympathetic modulation. In fact, it represents the oscillatory rhythmical properties of sympathetic discharge. The HF oscillatory component is synchronous with breathing, and it reflects cardiac parasympathetic modulation. The ratio between LF/HF components is related to the cardiac sympathovagal balance [27,28,29]. Regarding the SAPV, the absolute value of the LF component represents the sympathetic vascular modulation [30].

### 2.4. Symbolic Analysis

A nonlinear method to assess the complexity of cardiovascular regulatory mechanisms (HeartScope II; AMPS-LLC, New York, USA) was also used in the present study [31,32,33]. It is able to provide general information about sympathetic and parasympathetic modulation through a short period from HRV. The signal is decomposed into 3-beat sequence sets, spread over six levels and then classified into four families: 0V%, patterns with no variations; 1V%, patterns with one variation; 2LV%, patterns with two like and 2UV% patterns with two unlike variations. The 0V% pattern is a marker of cardiac sympathetic while 2LV% and 2UV% are markers of cardiac vagal modulation [32]. In this study, we applied symbolic analysis at the same RR interval of 200–300 beats used in the spectral analysis, taking into account each subject. The protocol challenges had its own rage of RR intervals. Regarding the SAPV, only the 0V% pattern represents the sympathetic vascular modulation.

### 2.5. Arterial Baroreflex Control

The cross-spectral analysis was performed by means of a bivariate autoregressive approach and the model order was fixed to 10 using the HeartScope II program (AMPS-LLC, New York, USA) [27]. This procedure allows the quantification of gain, coherence (K^2^), and phase (Φ) between different signal variabilities [4,34]. The gain of the transfer function quantifies the intensity of the response of the output signal (RRi) per unit of spontaneous change of the input signal (SAP). The function of K^2^ measures the degree of linear coupling between RRi and SAP at the same frequency in both variability signals, while the phase shift (Φ) measures the time lag or lead between the signals. In the present study, the arterial baroreflex control of HR was obtained in all cases in which the coherence index was significant (K^2^ > 0.5) and the phase shift in radians was negative (Φ < 0 radians, i.e., systolic arterial pressure changes precede R–R interval changes). The value of the arterial baroreflex control of HR is expressed in ms/mmHg [4,34].

### 2.6. Statistical Analysis

Statistical analyses were performed using the Sigma plot version 11.0. All data are presented as median and interquartile range (25th–75th percentile). For each continuous or discrete variable, the Lèvene and Shapiro–Wilk tests were used to assess the homogeneity and normality of distribution, respectively. In resting conditions, differences between types of electrical stimulation were tested by paired Student’s *t*-test. The relative responses (Ä%) of the cardiac and peripheral autonomic control during orthostatic change were also compared using the paired Student’s *t*-test. Wilcoxon signed-rank test was used when the data were not normally distributed. Probability values of *p* < 0.05 were considered statistically significant.

## 3. Results

The physical and hemodynamic characteristics of the subjects are shown in Table 1. Data of cardiac and peripheral autonomic control evaluated by linear and nonlinear tools in the resting condition during the baseline recording (Rest_tVNS off) or during tVNS application (Rest_tVNS on) are shown in Table 2. As to cardiac autonomic control, although tVNS significantly decreased heart rate (HR) (63 (60–66) vs. 66 (61–68) bpm, *p* < 0.01), no significant difference was observed in the relative contribution of the low-frequency and high-frequency components at spectral decomposition. By contrast, nonlinear analysis of the R–R interval showed a significant decrease in the frequency of the no variation pattern (0V%), a marker of cardiac sympathetic modulation (17 (5–20) vs. 18 (8–27) %, *p* < 0.01). Similarly, when analyzing the effects of tVNS on peripheral autonomic control, the marker of peripheral sympathetic modulation (0V % SAP) was significantly reduced (17 (13–30) vs. 36 (14–47) %, *p* < 0.01) (Table 2).

The effects of tVNS on arterial baroreflex control of HR are reported in Table 3. The respiratory rate was significantly coupled with only the HF component of the R–R interval (K^2^_HF_ > 85%), as indicated by a high degree of coherence between these variability signals in both conditions (Table 3). tVNS did not induce any significant change in the cardiorespiratory coupling and baroreflex control.

The autonomic variables evaluated by means of linear and nonlinear approaches were similar in the resting condition when compared between experimental sessions (rest_control vs. rest_tVNS off, supplementary Tables S1–S3).

**Figure 1:**
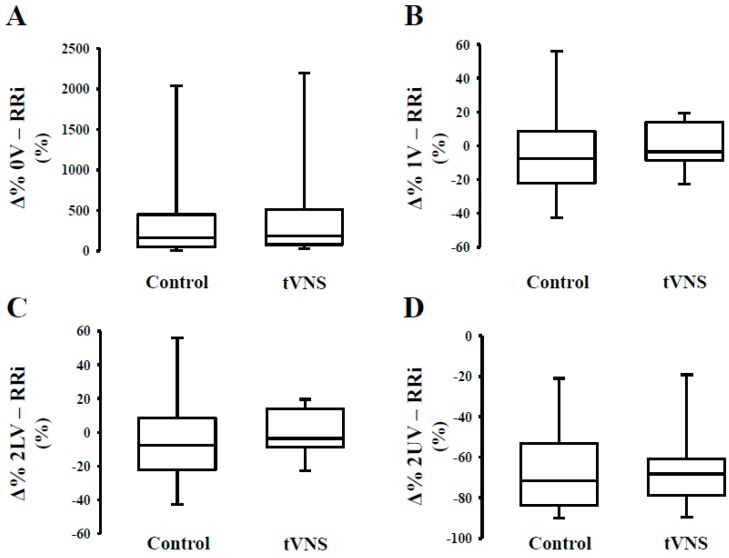
Response (Δ%) of cardiac autonomic control to orthostatic stress during transcutaneous vagus nerve stimulation in healthy subjects. Note that the orthostatic challenge promoted similar responses on cardiac sympathetic modulation (Panel **A**) and cardiac parasympathetic modulation (Panel **C** and **D**) independent of the stimulus (control or tVNS). Similar results are observed for 1V% (Panel **B**).

**Figure 2:**
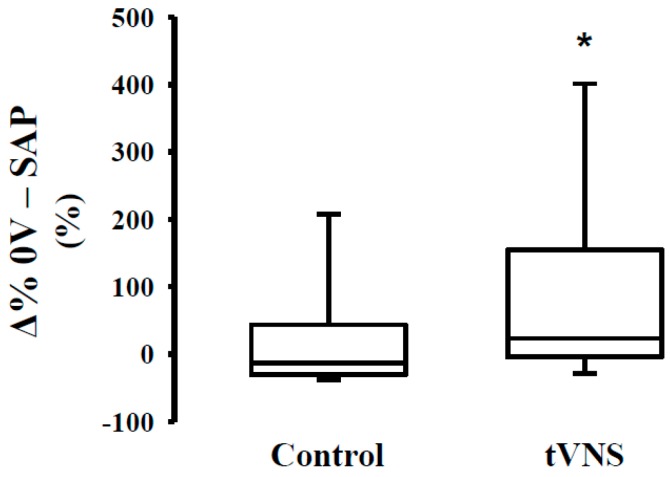
Response (Δ%) of peripheral autonomic control to orthostatic stress during transcutaneous vagus nerve stimulation in healthy subjects. Note that the response to orthostatic challenge during tVNS session was higher when compared to the control session. * = difference vs. control, *p* < 0.05.

As to the effects of the orthostatic maneuver, we observed a similar relative response (Ä%) during the control and tVNS sessions (Table 4). In Figure 1, we can observe that orthostatic stress increases cardiac sympathetic modulation (Figure 1, Panel A) and decreases cardiac parasympathetic modulation (Figure 1, Panel C,D), regardless of the stimulation. The response of the peripheral autonomic control to orthostatic stress evaluated by means of a nonlinear approach is shown in Figure 2. Interestingly, the responsivity of the peripheral sympathetic modulation to orthostatic stress during tVNS was significantly higher compared to the response on the control day (*p* = 0.03) (Figure 2).

## 4. Discussion

The main findings from the present study are that acute tVNS (1) reduces HR when compared to baseline, (2) decreases cardiac and peripheral sympathetic modulation in the rest condition, and, (3) increases the responsivity of the sympathetic vasomotor modulation to orthostatic change in young healthy subjects.

The vagal system plays a very important role in the regulation and homeostasis of several pathways. A few years ago, Tracey [35] described the so-called “inflammatory reflex,” i.e., a neural circuit that is elicited by cytokines and activates the vagus nerve to suppress the release of pro-inflammatory cytokines. A large amount of literature has shown that vagal control plays a very important regulatory role for different biological systems, from inflammation to immunity and from the endocrine to cardiovascular systems. In addition, as shown by the elegant study by Weber et al. [36], a low vagal tone was associated with altered post-stress recovery of different systems (cardiovascular, endocrine, and immune system), thus suggesting that vagal activity is a key homeostatic agent of body systems and vagal withdrawal is a risk factor for stress-related disorders [36].

In this study, we focused on the cardiovascular effects of a new and non-invasive technique able to stimulate and modulate vagus nerves in an innovative way.

The reduction in resting HR reveals a direct effect of tVNS with important clinical implication. In this study, we observed that tVNS promoted a decrease greater than 4% in the resting HR. This result may represent the result of direct parasympathetic vagal activity elicited by the auricular stimulation. However, the mechanism by which tVNS—that is, neuromodulation and not a simple stimulation of neural fibers—reduces HR still needs to be elucidated.

Increased HR is a marker of dysautonomia and a reduction in HR is one of the therapeutic targets in several cardiovascular diseases. However, a recent study [8] demonstrated that according to the parameters of tVNS, the HR effects are time dependent. In particular, by exploring a wide range of pulse widths and pulse frequencies during only one minute of stimulation, Badran et al. [37] showed that the optimal parameter for stimulating heart rate is 500 µs, 25 Hz. Since we used a pulse width of 200 µs and a pulse frequency of 25 Hz during 10 minutes of stimulation, we observed that these parameters were sufficient to promote a reduction in heart rate in healthy subjects. However, we cannot exclude that different stimulation parameters may also differently affect the cardiac and peripheral autonomic control. Thus, tVNS is a promising adjuvant therapy for a lot of drug-refractory disorders (e.g., refractory hypertension); however, further studies are required to validate this hypothesis.

In the present study, we found no difference in the spectral parameters of the cardiovascular variabilities under resting conditions. However, the symbolic analysis was able to identify a reduction in cardiac sympathetic modulation promoted by tVNS with no changes in cardiac parasympathetic modulation. Another important new finding in our study is that tVNS reduces the sympathetic vasomotor modulation in healthy individuals in the resting condition. These effects of tVNS on cardiac and peripheral sympathetic modulation may explain at least in part the reduction in heart rate and variability in systolic arterial pressure. As all spectral indexes are useful only under conditions characterized by reciprocal changes in sympathetic and parasympathetic modulations, this tool may not be able to detect subtle changes in one of the autonomic branches induced by the tVNS acutely [31,33]. In line with this conception, Guzzetti et al. proposed a nonlinear approach of HRV analysis (i.e., symbolic analysis) to quantify the prevalence of sympathetic or parasympathetic cardiac modulation in conditions in which the use of a linear HRV method is limited or even disputable [31].

As far as we know, this is the first study that evaluates the cardiac autonomic control during tVNS by means of a nonlinear approach in young healthy subjects. Symbolic analysis has the potential to detect nonreciprocal changes in sympathetic and parasympathetic modulation or reciprocal changes with different magnitudes [31]. A hallmark of the autonomic nervous system is its ability to induce variations on the target organ (i.e., heart, vessels). In the absence of autonomic dysfunction, the tonic and phasic activities of cardiovascular variabilities are coupled and synchronized in healthy individuals [2].

We also investigated the effects of tVNS on arterial baroreflex control in both modulation ranges. In contrast to the findings of Antonino et al. [21], we did not observe an increase in the gain of arterial baroreflex control of HR during tVNS in healthy subjects under the rest condition. Thus, we believe that the mechanism involved in the reduction of cardiac and peripheral sympathetic modulation promoted by tVNS was a direct effect on NTS. This hypothesis is supported by recent findings in the neuroimaging area. A recent study demonstrated that stimulation at the *cymba conchae* produced a significantly stronger activation in the NTS when compared with other locations in the ear [38]. A classic concept in autonomic neuroscience is that NTS is the first synaptic station of the afferent projections in the central nervous system, and it plays a key role in the modulation of the autonomic efferent activity directed to the cardiovascular system [39]. In order to produce a proper autonomic response, information from several relay stations must be processed at the NTS level, where all the projections of many and complex neural networks are processed and then organized by means of hierarchical levels into different reflex responses [2]. We observed significant changes in the sympathetic and not vagal modulation during tVNS in healthy subjects. Due to the absence of autonomic dysfunction, our data reveal the effect of tVNS on directly modulating the cardiac and peripheral sympathetic branch. Based on these findings, we could speculate that the stimulation of the auricular branch of the vagus nerve may activate and modulate not only efferent but also afferent fibers in physiological conditions. Thus, we hypothesized that tVNS acts as a neuro-modulator of ANS, directly at the brainstem level, possibly through a direct action on NTS. Other studies are warranted to confirm this hypothesis.

Besides the resting condition, the autonomic nervous system dynamically adjusts the functions of the cardiovascular system to ensure the adequate levels of cardiac output to meet the perfusion and metabolic requirements of the peripheral organ systems [1,2]. In this sense, several authors have emphasized that the autonomic nervous system should also be evaluated under physiological stress with the aim of examining the complexities of neural regulation without artificially isolating the influence of the autonomic branches [31,33,34].

This dynamic interaction during orthostatic change has been widely used in the investigation of possible autonomic dysfunctions [40,41,42]. Thus, we believe that evaluation of the ANS response to physiological stress during tVNS can be recommended, but further ad hoc studies are needed especially in patients using drugs that act on ANS. As tVNS reduces the adrenergic efferent drive at rest, an increased response of the sympathetic vasomotor modulation to orthostatic challenge is an expected physiological response. It reveals the integrity and ability of the system to adapt and properly react to physiological stress. These findings have important clinical relevance.

This study has strengths and limitations. The major limitation of the present study is that these findings cannot be extrapolated to other populations such as older and/or diseased subjects. Due to the small sample size, another limitation of this study is that the non-Gaussian distributed variables make the use of statistical parametric tests unfeasible. However, it has several strengths. Firstly, we assessed the acute effects of tVNS in resting conditions and in response to an orthostatic challenge and this allowed us to evaluate the dynamics of cardiac and peripheral autonomic control before and after physiological stress under acute effects of tVNS. Finally, we used different tools to provide complementary information of ANS (i.e., spectral and symbolic analysis).

## 5. Conclusions

This study demonstrated that transcutaneous vagus nerve stimulation reduces the HR and affects cardiac and peripheral autonomic regulation, sympathetic modulation directed to the heart and the vessels. Thus, these findings suggest that evaluation of the autonomic nervous system response to orthostatic stress during tVNS can be recommended mainly in screening for possible adverse cardiovascular events.

## Figures and Tables

**Table 1:** Physical and hemodynamic characteristics of the study sample.

Characteristic	
***Physical***	
Gender (male/female)	5/8
Age (years)	26 (23–31)
Body weight (kg)	63 (55–75)
BMI (kg/m^2^)	22 (20–24)
***Hemodynamics***	
SAP (mmHg)	100 (96–113)
DAP (mmHg)	70 (60–76)
MAP (mmHg)	80 (72–88)
HR (beats/min)	66 (60–73)

Median (25th–75th). BMI = body mass index; SAP = systolic arterial pressure; DAP = diastolic arterial pressure; MAP = mean arterial pressure; HR = heart rate.

**Table 2:** Baseline measures of cardiac and peripheral autonomic control evaluated by linear and nonlinear approaches.

	Rest_tVNS off (*n* = 13)	Rest_tVNS on (*n* = 13)	*P*
**Linear Analysis**			
***R–R interval***			
HR (beats/min)	66 (61–68)	63 (60–66)	<0.01
Variance (ms^2^)	2572 (1494–4291)	2549 (1717–3331)	0.54
VLF abs. (ms^2^)	1146 (580–1475)	1132 (485–1439)	0.98
LF n.u. (%)	41 (19–62)	53 (28–63)	0.27
HF n.u. (%)	57 (35–71)	46 (35–69)	0.45
LF/HF	0.7 (0.3–1.9)	1.2 (0.4–1.8)	0.50
***Systolic Arterial Pressure***			
Variance (mmHg^2^)	19 (8–27)	13 (6–19)	<0.01
LF abs. (mmHg^2^)	1.0 (0.6–2.2)	1.4 (0.6–2.8)	0.37
***Respiratory Rate***			
HF (Hz)	0.29 (0.20–0.33)	0.29 (0.26–0.31)	0.73
	**Nonlinear Analysis**		
***R–R interval patterns***			
0V (%)	18 (8–27)	17 (5–20)	<0.01
1V (%)	45 (44–52)	49 (43–52)	0.24
2LV (%)	11 (7–22)	12 (10–20)	0.95
2UV (%)	21 (11–32)	25 (16–33)	0.31
***Systolic Arterial Pressure Pattern***			
0V (%)	36 (14–47)	17 (13–30)	<0.01

Median (25th–75th). tVNS = transcutaneous vagus nerve stimulation; HR = heart rate; VLF = very low frequency; LF = low frequency; HF = high frequency; LF/HF = sympathovagal balance; n.u. = normalized unit, ω = central frequency.

**Table 3:** Baseline measures of cardiorespiratory coupling and baroreflex control evaluated by transfer function analysis.

	Rest_tVNS off (*n* = 13)	Rest_tVNS on (*n* = 13)	*P*
***RESP-RRi***			
K^2^_HF_ (%)	93 (88–98)	95 (90–98)	0.55
***SAP-RRi***			
Gain_LF_ (ms/mmHg)	12 (9–27)	15 (10–26)	0.37
Gain_HF_ (ms/mmHg)	17 (11–36)	18 (14–36)	0.99
K^2^_LF_ (%)	75 (65–84)	73 (59–85)	0.72
K^2^_HF_ (%)	96 (89–97)	95 (87–98)	0.51

Median (25^th^–75th). tVNS = transcutaneous vagus nerve stimulation; RESP = respiration; RRi = R–R interval; SAP = systolic arterial pressure; K^2^ = coherence; LF = low frequency band; HF = high frequency band.

**Table 4:** Response (Δ%) of cardiac and peripheral autonomic control evaluated by a linear method using an autoregressive model.

	Tilt_Control (*n* = 13)	Tilt_tVNS (*n* = 13)	*P*
**LINEAR ANALYSIS**			
***R–R interval***			
RRi (%)	−21 (−29–−13)	−23 (−29–−17)	0.07
Variance (%)	−24 (−51–25)	−42 (−68–40)	0.46
VLF abs. (%)	−28 (−87–15)	−28 (−72–85)	0.21
LF n.u. (%)	63 (32–131)	52 (30–177)	0.89
HF n.u. (%)	−70 (−84–−57)	−67 (−87–−60)	0.71
LF/HF (%)	669 (226–1017)	737 (253–1456)	0.95
**Systolic Arterial Pressure**			
Variance (%)	68 (0–102)	93 (39–137)	0.50
LF abs. (%)	229 (151–775)	404 (112–813)	0.68
**Respiratory Rate**			
ù HF (%)	−5 (−14–9)	6 (1–12)	0.08

Median (25th–75th). tVNS = transcutaneous vagus nerve stimulation; RRi = R–R interval; VLF = very low frequency; LF = low frequency; HF = high frequency; LF/HF = sympathovagal balance; n.u. = normalized unit, ω = central frequency.

## Supplementary Materials

The following are available online at https://www.mdpi.com/2077-0383/8/4/496/s1, Table S1: Baseline measures of spectral parameters calculated by linear method using autoregressive model, Table S2. Baseline measures of dynamics patterns calculated by nonlinear method using symbolic analysis, Table S3. Baseline measures of transfer function calculated by bivariate autoregressive model.

## References

[1] Montano N., Tobaldini E., Porta A., Chouker A. (2012). The Autonomic Nervous System. Stress Challenges and Immunity in Space: From Mechanisms to Monitoring and Preventive Strategies.

[2] Toschi-Dias E., Rondon M.U.P.B., Cogliati C., Paolocci N., Tobaldini E., Montano N. (2017). Contribution of Autonomic Reflexes to the Hyperadrenergic State in Heart Failure. Front. Neurosci..

[3] Ichinose M., Saito M., Fujii N., Kondo N., Nishiyasu T. (2006). Modulation of the control of muscle sympathetic nerve activity during severe orthostatic stress. J. Physiol..

[4] Montano N., Ruscone T.G., Porta A., Lombardi F., Pagani M., Malliani A. (1994). Power spectrum analysis of heart rate variability to assess the changes in sympathovagal balance during graded orthostatic tilt. Circulation.

[5] Mancia G., Grassi G. (2014). The Autonomic Nervous System and Hypertension. Circ. Res..

[6] Guzzetti S., Mennini T., Cagnotto A., Di Biasi P., Scrofani R., Mezzetti S., Cogliati C., Paglia S., Malliani A. (1998). Myocardial β-adrenergic and muscarinic receptor density in cardiac pressure or volume overload. J. Mol. Cell. Cardiol..

[7] Barretto A.C., Santos A.C., Munhoz R., Rondon M.U., Franco F.G., Trombetta I.C., Roveda F., De Matos L.N., Braga A.M., Middlekauff H.R. (2009). Increased muscle sympathetic nerve activity predicts mortality in heart failure patients. Int. J. Cardiol..

[8] La Rovere M.T., Pinna G.D., Maestri R., Mortara A., Capomolla S., Febo O., Ferrari R., Franchini M., Gnemmi M., Opasich C. (2003). Short-term heart rate variability strongly predicts sudden cardiac death in chronic heart failure patients. Circulation.

[9] Juraschek S.P., Daya N., Appel L.J., Miller E.R., McEvoy J.W., Matsushita K., Ballantyne C.M., Selvin E. (2018). Orthostatic Hypotension and Risk of Clinical and Subclinical Cardiovascular Disease in Middle-Aged Adults. J. Am. Heart Assoc..

[10] Folino A.F., Tokajuk B., Porta A., Romano S., Cerutti S., Volta S.D. (2005). Autonomic modulation and clinical outcome in patients with chronic heart failure. Int. J. Cardiol..

[11] Ponikowski P., Voors A.A., Anker S.D., Bueno H., Cleland J.G., Coats A.J., Falk V., González-Juanatey J.R., Harjola V.P., Jankowska E.A. (2016). 2016 ESC Guidelines for the diagnosis and treatment of acute and chronic heart failure: The Task Force for the diagnosis and treatment of acute and chronic heart failure of the European Society of Cardiology (ESC). Developed with the special contribution of the Heart Failure Association (HFA) of the ESC. Eur. J. Heart Fail..

[12] De Matos L.D., Gardenghi G., Rondon M.U., Soufen H.N., Tirone A.P., Barretto A.C., Brum P.C., Middlekauff H.R., Negrão C.E. (2004). Impact of 6 months of therapy with carvedilol on muscle sympathetic nerve activity in heart failure patients. J. Card. Fail..

[13] Toschi-Dias E., Trombetta I.C., Silva V.J.D., Maki-Nunes C., Cepeda F.X., Alves M.J.N.N., Carvalho G.L., Drager L.F., Lorenzi-Filho G., Negrão C.E. (2019). Diet associated with exercise improves baroreflex control of sympathetic nerve activity in metabolic syndrome and sleep apnea patients. Sleep Breath..

[14] Roveda F., Middlekauff H.R., Rondon M.U.P.B., Reis S.F., Souza M., Nastari L., Barretto A.C.P., Krieger E.M., Negrão C.E. (2003). The effects of exercise training on sympathetic neural activation in advanced heart failure: A randomized controlled trial. J. Am. Coll. Cardiol..

[15] Bernardi L., Sleight P., Bandinelli G., Cencetti S., Fattorini L., Wdowczyc-Szulc J., Lagi A. (2001). Effect of rosary prayer and yoga mantras on autonomic cardiovascular rhythms: Comparative study. BMJ.

[16] Toschi-Dias E., Tobaldini E., Solbiati M., Costantino G., Sanlorenzo R., Doria S., Irtelli F., Mencacci C., Montano N. (2017). Sudarshan Kriya Yoga improves cardiac autonomic control in patients with anxiety-depression disorders. J. Affect. Disord..

[17] Boon P., De Cock E., Mertens A., Trinka E. (2018). Neurostimulation for drug-resistant epilepsy: A systematic review of clinical evidence for efficacy, safety, contraindications and predictors for response. Curr. Opin. Neurol..

[18] Franzini A., Messina G., Marras C., Savino M., Miniati M., Bugiani O., Broggi G. (2008). Hamilton Rating Scale for Depression-21 Modifications in Patients with Vagal Nerve Stimulation for Treatment of Treatment-Resistant Depression: Series Report. Neuromodulation Technol. Neural Interface.

[19] Frangos E., Ellrich J., Komisaruk B.R. (2015). Non-invasive Access to the Vagus Nerve Central Projections via Electrical Stimulation of the External Ear: FMRI Evidence in Humans. Brain Stimul..

[20] Kraus T., Kiess O., Hösl K., Terekhin P., Kornhuber J., Förster C. (2013). CNS BOLD fMRI Effects of Sham-Controlled Transcutaneous Electrical Nerve Stimulation in the Left Outer Auditory Canal—A Pilot Study. Brain Stimul..

[21] Antonino D., Teixeira A.L., Maia-Lopes P.M., Souza M.C., Sabino-Carvalho J.L., Murray A.R., Deuchars J., Vianna L.C. (2017). Non-invasive vagus nerve stimulation acutely improves spontaneous cardiac baroreflex sensitivity in healthy young men: A randomized placebo-controlled trial. Brain Stimul..

[22] Clancy J.A., Mary D.A., Witte K.K., Greenwood J.P., Deuchars S.A., Deuchars J. (2014). Non-invasive Vagus Nerve Stimulation in Healthy Humans Reduces Sympathetic Nerve Activity. Brain Stimul..

[23] De Couck M., Cserjesi R., Caers R., Zijlstra W.P., Widjaja D., Wolf N., Luminet O., Ellrich J., Gidron Y. (2017). Effects of short and prolonged transcutaneous vagus nerve stimulation on heart rate variability in healthy subjects. Auton. Neurosci..

[24] Heart Rate Variability (1996). Standards of measurement, physiological interpretation, and clinical use. Task Force of the European Society of Cardiology and the North American Society of Pacing and Electrophysiology. Circulation.

[25] Kreuzer P.M.M., Landgrebe M., Husser O., Resch M., Schecklmann M., Geisreiter F., Poeppl T.B., Prasser S.J., Hajak G., Langguth B. (2012). Transcutaneous Vagus Nerve Stimulation: Retrospective Assessment of Cardiac Safety in a Pilot Study. Front. Psychol..

[26] Kreuzer P.M., Landgrebe M., Resch M., Husser O., Schecklmann M., Geisreiter F., Poeppl T.B., Prasser S.J., Hajak G., Rupprecht R. (2014). Feasibility, Safety and Efficacy of Transcutaneous Vagus Nerve Stimulation in Chronic Tinnitus: An Open Pilot Study. Brain Stimul..

[27] Barbic F., Heusser K., Marchi A., Zamunér A.R., Gauger P., Tank J., Jordan J., Diedrich A., Robertson D., DiPaola F. (2015). Cardiovascular Parameters and Neural Sympathetic Discharge Variability before Orthostatic Syncope: Role of Sympathetic Baroreflex Control to the Vessels. Physiol. Meas..

[28] Malliani A., Pagani M., Lombardi F., Cerutti S. (1991). Cardiovascular neural regulation explored in the frequency domain. Circulation.

[29] Montano N., Porta A., Cogliati C., Costantino G., Tobaldini E., Casali K.R., Iellamo F. (2009). Heart rate variability explored in the frequency domain: A tool to investigate the link between heart and behavior. Neurosci. Biobehav. Rev..

[30] Stauss H.M. (2007). Power spectral analysis in mice: What are the appropriate frequency bands?. Am. J. Physiol. Integr. Comp. Physiol..

[31] Guzzetti S., Borroni E., Garbelli P.E., Ceriani E., Della Bella P., Montano N., Cogliati C., Somers V.K., Malliani A., Porta A. (2005). Symbolic Dynamics of Heart Rate Variability: A Probe to Investigate Cardiac Autonomic Modulation. Circulation.

[32] Porta A., Guzzetti S., Montano N., Furlan R., Pagani M., Malliani A., Cerutti S. (2001). Entropy, entropy rate, and pattern classification as tools to typify complexity in short heart period variability series. IEEE Trans. Biomed. Eng..

[33] Porta A., Tobaldini E., Guzzetti S., Furlan R., Montano N., Gnecchi-Ruscone T. (2007). Assessment of cardiac autonomic modulation during graded head-up tilt by symbolic analysis of heart rate variability. Am. J. Physiol. Circ. Physiol..

[34] Toschi-Dias E., Trombetta I.C., Da Silva V.J.D., Maki-Nunes C., Cepeda F., Alves M.-J.N.N., Drager L.F., Lorenzi-Filho G., Negrão C.E., Rondon M.U.P.B. (2013). Time delay of baroreflex control and oscillatory pattern of sympathetic activity in patients with metabolic syndrome and obstructive sleep apnea. Am. J. Physiol. Circ. Physiol..

[35] Tracey K.J. (2002). The inflammatory reflex. Nature.

[36] Weber C.S., Thayer J.F., Rudat M., Wirtz P.H., Zimmermann-Viehoff F., Thomas A., Perschel F.H., Arck P.C., Deter H.C. (2010). Low vagal tone is associated with impaired post stress recovery of cardiovascular, endocrine, and immune markers. Graefe’s Arch. Clin. Exp. Ophthalmol..

[37] Badran B.W., Mithoefer O.J., Summer C.E., Labate N.T., Glusman C.E., Badran A.W., Devries W.H., Summers P.M., Austelle C.W., McTeague L.M. (2018). Short trains of transcutaneous auricular vagus nerve stimulation (taVNS) have parameter-specific effects on heart rate. Brain Stimul..

[38] Yakunina N., Kim S.S., Nam E.C. (2017). Optimization of Transcutaneous Vagus Nerve Stimulation Using Functional MRI. Neuromodulation.

[39] Machado B., Mauad H., Chianca D.A., Haibara A., Colombari E. (1997). Autonomic processing of the cardiovascular reflexes in the nucleus tractus solitarii. Braz. J. Med Biol. Res..

[40] Furlan R., Colombo S., Perego F., Atzeni F., Diana A., Barbic F., Porta A., Pace F., Malliani A., Sarzi-Puttini P. (2005). Abnormalities of cardiovascular neural control and reduced orthostatic tolerance in patients with primary fibromyalgia. J. Rheumatol..

[41] Miyamoto S., Fujita M., Sekiguchi H., Okano Y., Nagaya N., Ueda K., Tamaki S.-I., Nohara R., Eiho S., Sasayama S. (2001). Effects of posture on cardiac autonomic nervous activity in patients with congestive heart failure. J. Am. Coll. Cardiol..

[42] Radaelli A., Perlangeli S., Cerutti M.C., Mircoli L., Mori I., Boselli L., Bonaita M., Terzoli L., Candotti G., Signorini G. (1999). Altered blood pressure variability in patients with congestive heart failure. J. Hypertens..

