# Supplementary material for: Cardiac and Peripheral Autonomic Responses to Orthostatic Stress During Transcutaneous Vagus Nerve Stimulation in Healthy Subjects
Source: J Clin Med. 2019 Apr 11;8(4):496. doi: 10.3390/jcm8040496 (PMC6517949; doi:10.3390/jcm8040496)
Supplement: Supplementary file 1 [file jcm-08-00496-s001.pdf]

Table S1. Baseline measures of spectral parameters calculated by linear method using autoregressive model

	**Rest_Control (n=13)**	**Rest_tVNS off (n=13)**	***P***
R-R interval			
HR (beats/min)	67 [60 – 72]	66 [61 – 68]	0.25
Variance (ms^2)	1690 [1336 – 3725]	2572 [1494 – 4291]	0.24
VLF abs. (ms^2)	758 [490 – 1262]	1146 [580 – 1475]	0.30
LF n.u. (%)	46 [30 – 66]	41 [19 – 62]	0.21
HF n.u. (%)	49 [33 – 67]	57 [35 – 71]	0.21
LF/HF	1.0 [0.5 – 2.1]	0.7 [0.3 – 1.9]	0.23
Systolic Arterial Pressure			
Variance ($mmHg^2$)	20 [13 – 30]	19 [8 – 27]	0.26
LF abs. ($mmHg^2$)	1.8 [0.6 – 4.1]	1.0 [0.6 – 2.2]	0.28
Respiratory Rate			
ω HF (Hz)	0.31 [0.24 – 0.33]	0.29 [0.20 – 0.33]	0.40
HF n.u. (%)	94 [85 – 96]	93 [89 – 96]	0.95

Median [25th-75th]; tVNS = transcutaneous vagal nerve stimulation. VLF = very low frequency; LF = low frequency; HF = high frequency; LF/HF = sympathovagal balance; n.u. = normalized unit.

Table S2. Baseline measures of dynamics patterns calculated by nonlinear method using symbolic analisys

	Rest_Control (n=13)	Rest_tVNS off (n=13)	*P*
RRi Patterns			
0V (%)	14 [8 – 25]	18 [8 – 27]	0.63
1V (%)	49 [46 – 54]	45 [44 – 52]	0.18
2LV (%)	11 [8 – 19]	11 [7 – 22]	0.84
2UV (%)	21 [11 – 26]	21 [11 – 32]	0.75
SAP Patterns			
0V (%)	35 [21 – 44]	36 [14 – 47]	0.58

Median [25th-75th]. tVNS = transcutaneous vagal nerve stimulation; RRi= R-R interval; SAP = systolic arterial pressure.

Table S3. Baseline measures of transfer function calculated by bivariate autoregressive model

	**Rest_Control (n=13)**	**Rest_tVNS off (n=13)**	***P***
RESP-RRi			
K^2_{LF} (%)	14 [1 – 29]	27 [19 – 38]	0.41
K^2_{HF} (%)	96 [92 – 97]	93 [88 – 98]	0.02
SAP-RRi			
$Gain_{LF}$ (ms/mmHg)	14 [8 – 18]	12 [9 – 27]	0.84
$Gain_{HF}$ (ms/mmHg)	19 [12 – 35]	17 [11 – 36]	0.45
K^2_{LF} (%)	62 [48 – 86]	75 [65 - 84]	0.60
K^2_{HF} (%)	96 [93 – 98]	96 [89 – 97]	0.91

Median [25th-75th]. tVNS = transcutaneous vagal nerve stimulation; RRi= R-R interval; RESP = respiration; SAP = systolic arterial pressure; LF = low frequency; HF = high frequency; K^2 = coherence.
